# Survey of extrachromosomal circular DNA derived from plant satellite repeats

**DOI:** 10.1186/1471-2229-8-90

**Published:** 2008-08-22

**Authors:** Alice Navrátilová, Andrea Koblížková, Jiří Macas

**Affiliations:** 1 Biology Centre ASCR, Institute of Plant Molecular Biology, Branišovská 31, České Budějovice, CZ-37005, Czech Republic

## Abstract

**Background:**

Satellite repeats represent one of the most dynamic components of higher plant genomes, undergoing rapid evolutionary changes of their nucleotide sequences and abundance in a genome. However, the exact molecular mechanisms driving these changes and their eventual regulation are mostly unknown. It has been proposed that amplification and homogenization of satellite DNA could be facilitated by extrachromosomal circular DNA (eccDNA) molecules originated by recombination-based excision from satellite repeat arrays. While the models including eccDNA are attractive for their potential to explain rapid turnover of satellite DNA, the existence of satellite repeat-derived eccDNA has not yet been systematically studied in a wider range of plant genomes.

**Results:**

We performed a survey of eccDNA corresponding to nine different families and three subfamilies of satellite repeats in ten species from various genera of higher plants (*Arabidopsis*, *Oryza*, *Pisum*, *Secale*, *Triticum *and *Vicia*). The repeats selected for this study differed in their monomer length, abundance, and chromosomal localization in individual species. Using two-dimensional agarose gel electrophoresis followed by Southern blotting, eccDNA molecules corresponding to all examined satellites were detected. EccDNA occurred in the form of nicked circles ranging from hundreds to over eight thousand nucleotides in size. Within this range the circular molecules occurred preferentially in discrete size intervals corresponding to multiples of monomer or higher-order repeat lengths.

**Conclusion:**

This work demonstrated that satellite repeat-derived eccDNA is common in plant genomes and thus it can be seriously considered as a potential intermediate in processes driving satellite repeat evolution. The observed size distribution of circular molecules suggests that they are most likely generated by molecular mechanisms based on homologous recombination requiring long stretches of sequence similarity.

## Background

Higher plant genomes contain considerable amounts of satellite repeats which make up to 20% of nuclear DNA in some species [1]. Satellite repeats occur in a genome as continuous arrays of tandemly arranged basic repeated units (monomers). Although the monomers are usually only tens to hundreds of nucleotides long, they can accumulate into millions of copies, forming megabase-sized clusters distinguishable as heterochromatic regions on mitotic chromosomes or in interphase nuclei. Satellite repeats undergo rapid evolutionary changes of their sequences and abundance, leading to the frequent occurrence of genus- or species-specific families of satellite DNA [2-5]. Contrary to this diversification observed between various taxa, the repeat monomers are usually well-homogenized within a species. This process of intra-specific sequence homogenization, generally referred to as 'concerted evolution', is supposed to arise from a concurrent action of various molecular mechanisms including unequal crossing-over and gene conversion [6,7]. Although some of these mechanisms have been characterized in detail, their overall contribution to satDNA evolution remains elusive. Moreover, theoretical models and computer simulations suggest that these mechanisms alone cannot account for efficient amplification and long-term persistence of satellites within genomes [8-10]. Therefore, it is supposed that other processes capable of efficient sequence amplification probably act on satDNA. It has been proposed that they involve extrachromosomal circular DNA (eccDNA) molecules, arising from intra-strand recombination between monomers within satellite arrays and subsequently serving as a template for rolling-circle replication. This process would result in the synthesis of linear DNA fragments composed of multiple copies of the circular template molecules and their reintegration into the genome, thus providing an efficient mechanism for amplification and eventual sequence homogenization of satDNA.

Although eccDNA has been reported from a wide range of eukaryotic organisms including yeast, *Drosophila*, *Xenopus*, mouse and human [11-14], there are only a few studies focusing on its formation from satellite repeats [13-15]. In plants, eccDNA derived from centromeric repeats in *Arabidopsis *[16] and repetitive element Bdm29 in *Brachycome dichromosomatica *[17] have been detected. In spite of this progress in eccDNA research, its formation from a wider range of plant satellite repeats and different species has not been studied so far. Consequently, there is only a little known about the structure of circular DNA molecules in plant genomes and mechanisms of their formation. There is also an interesting question concerning the role of eccDNA in the evolution of monomer size of satellite repeats. Similar to other groups of eukaryotes, plant satellites show a clear preference for monomer sizes in ranges between 135 – 195 bp and their multiples [18]. Although the correspondence of this length with the length of DNA wrapped around nucleosome particles has been pointed out [19,20], there is no mechanism known to explain this phenomenon. It has been demonstrated that nucleosomes constrain accessibility of enzymatic apparatus to certain regions of associated DNA [21]. Thus, recombination-based sequence homogenization or excision of eccDNA may be more frequent in more accessible regions (e.g. nucleosome linkers), leading to the emergence of the nucleosome-sized repeated units.

In this study, we addressed some of the questions raised above by investigating the occurrence and properties of eccDNA molecules derived from satellite repeats in a range of species from three genera of higher plants (Fabaceae, Poaceae, Brassicaceae). The repeats to be studied were selected based on their various monomer lengths and eventual presence of higher-order repeats, in order to follow the importance of these properties for formation and size of eccDNA molecules. Our results demonstrated that formation of eccDNA from satellite repeats is a common phenomenon in higher plants, and that it is strongly dependent on sequence similarity.

## Results

**Figure 1:**
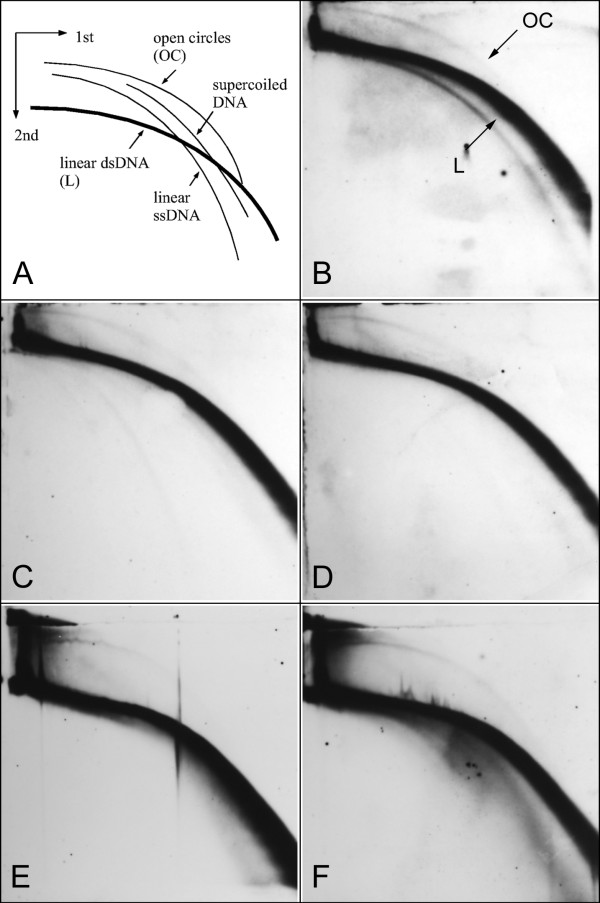
**Detection of eccDNA molecules derived from *Vicia faba *FokI repeats in different tissues and under stress conditions**. (A) Schematic outline of the migration patterns of linear and circular DNA forms on 2-D gel electrophoresis. (B-F) Detection of eccDNA in genomic DNA samples of *V. faba *separated on 2-D electrophoresis (0.4% and 1% agarose), Southern blotted and hybridized with FokI-derived probe. The DNA was isolated from root meristems (B), young (C) or old leaves (D), and from wounded leaves one (E) and two (F) days after the treatment.

We employed two-dimensional (2-D) agarose gel electrophoresis [22] followed by Southern blot hybridization to examine the presence of eccDNA in extracts of total genomic DNA from selected plant species. In addition to separating DNA molecules based on their size, 2-D electrophoresis also allows resolution based on their structure, resulting in formation of separated arcs on the gel representing linear and various conformations of circular DNA molecules (Fig. 1A). In our initial experiments we performed 2-D electrophoresis in 0.4% agarose in the first dimension, and in 1% agarose supplemented with ethidium bromide in the second dimension, which provided efficient resolution of linear and circular molecules from over 9 kb down to 3 kb as assessed from migration of linear and supercoiled or open circular marker molecules (not shown). These conditions were then used to analyze DNA samples of *Vicia faba*, a species containing highly abundant satellite repeat FokI [23,24] (Table 1). A strong arc of linear DNA and a weaker one corresponding to open circular molecules were revealed after hybridization with FokI probe. We used this species further for investigating FokI eccDNA levels in different tissues including young/developing or old leaves and root meristems (Fig. 1B–D), and also in leaf tissues stressed by wounding (Fig. 1E–F). However, we did not observe significant differences in eccDNA signals between these samples, suggesting that there are comparable levels of circular molecules derived from FokI satellite in all investigated *V. faba *tissues and that these levels are not significantly affected by stress conditions. As only open circular molecules were detected and there was no hybridization signal corresponding to covalently closed circular DNA, we tested whether this could be a result of damaging closed circles during DNA isolation. However, including supercoiled control plasmid DNA into tissue samples at the beginning of the procedure revealed that the isolation does not lead to significant nicking or other degradation of this DNA, and thus that open circles are the predominant form of satellite eccDNA in the examined tissues.

**Table 1:** Satellite repeats used in this study

**Satellite repeat family**	**Species**	**Monomer length (bp)**	**Location^(a)^**	**Copies/haploid genome (1C)**	**References**	**EccDNA size**	**Figure**
Afa	*Triticum aestivum*	340	P, I, T	30,000	[49-51]	(340)n	3C
AT-180	*Arabidopsis thaliana*	179	C, P	> 55,000	[52,53]	(179)n	
IGS-like	*Vicia sativa*	173	I	10,000 – 100,000	[47]	(173)n	3D
CentO	*Oryza sativa*	155	C, P	44,400	[54,55]	(155)n	3E
Sc119	*Secale cereale*	118	I, T	1,500,000	[56]	(118)n	3A, B
VicTR-A	*Vicia narbonensis*	69	I, T	100,000 – 1,000,000	[3,57]	(138)n	2
VicTR-A_c666	*Vicia pannonica*	69/138	n.d.^(b)^	n.d.^(b)^	this publication	continuous arc	3L
VicTR-A_c653	*Vicia pannonica*	180	n.d.^(b)^	n.d.^(b)^	this publication	(180)n	3K
FokI	*Vicia faba*	59	I	5,400,000 – 21,000,000	[24]	continuous arc	3F
PisTR-B	*Pisum sativum*	50	P, T	380,000	[58,59]	continuous arc	3G
VicTR-B	*Vicia grandiflora*	38	I, T	1,000,000 – 5,000,000	[3]	continuous arc	3H
VicTR-B_VG-V	*Vicia grandiflora*	186	T	n.d.	[26]	(186)n	3I
VicTR-B	*Vicia sativa*	38	I	1,000,000 – 5,000,000	[3]	continuous arc	

^(a) ^Positions of signals on chromosomes. C, centromeric; P, pericentromeric; I, intercalary; T, (sub-)telomeric.

^(b) ^VicTR-A family in V. *pannonica *is located in (sub-)telomeric chromosome regions [3], but chromosome localization and copy numbers of its specific sequence variants represented by the clones c666 and c653 were not determined.

**Figure 2:**
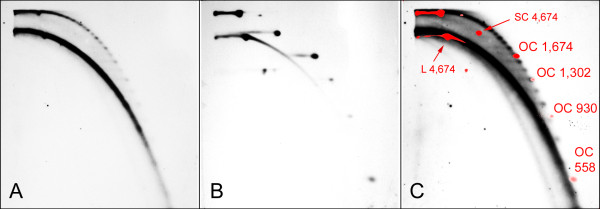
**Improved detection and size determination of VicTR-A repeats in *Vicia narbonensis***. EccDNA detection in *V. narbonensis *genomic DNA sample pre-treated with Plasmid-safe DNase and mung bean nuclease and resolved on 2-D electrophoresis using 0.7% and 2% agarose gels. Prior to electrophoresis, the sample was mixed with a set of circular DNA markers. The Southern blot of the gel was hybridized with VicTR-A probe (A), and then reprobed with the lambda DNA used to visualize DNA markers (B). Panel C shows superposition of signals from VicTR-A nad marker probes and gives the length of the open circle (OC), supercoiled (SC) and linear (L) markers (the lengths are in base pairs). VicTR-A signals on panel C are enhanced by longer exposure compared to panel A in order to reveal spots of the shortest circular molecules.

Following these initial experiments, we performed a detailed survey of the presence of eccDNA derived from nine satellite repeats and three subfamilies in ten plant species (Table 1). However, as eccDNA was found to occur at relatively low levels, we improved our isolation protocol by including treatment with Plasmid-safe ATP-dependent DNAse, which specifically degrades linear DNA while leaving covalently closed and nicked circular molecules intact. Removing the majority of linear fragments by this treatment allowed loading of equivalents of up to 240 μg of original undigested genomic DNA onto the gel. We also increased the discriminatory power of the technique towards efficient resolution of short circular molecules in order to exactly determine size differences of circular molecules derived from satellites with shorter monomers. This was achieved by increasing agarose concentrations to 0.7% and 2% in the first and second dimension, respectively, which led to improved resolution of circular DNA from linear fragments (down to 500 bp) and to resolving circular molecules differing by at least 90 bp. Size estimations of eccDNA molecules were done using a mixture of specifically designed markers including open circular molecules produced by Cre/lox recombination and nickase treatment of larger plasmid templates (see Methods for details). This is demonstrated in Figure 2, showing detection of VicTR-A satellite in *V. narbonensis*. Although the size of VicTR-A monomers is 69 bp, its eccDNA was found to occur in size steps of about 140 bp, thus corresponding to multiples of 138 bp. Moreover, there was an alternating pattern of stronger and weaker spots, suggesting more frequent excision of circular molecules differing in multiples of 276 bp.

**Figure 3:**
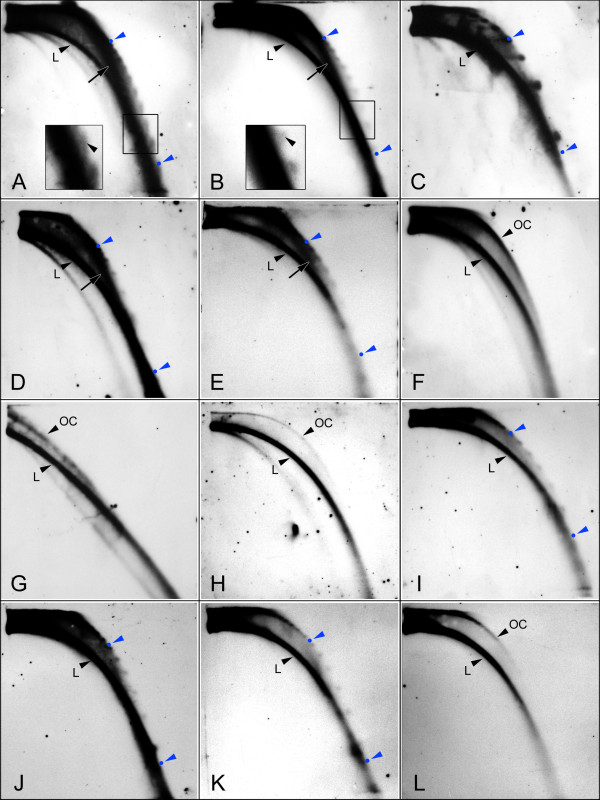
**Survey of eccDNA derived from various plant satellites**. Samples of genomic DNA enriched for circular DNA were separated on 0.7% and 2% 2-D gels, blotted and hybridized with satellite-specific probes. The arcs corresponding to linear (L) and open circle (OC) DNA are indicated with black arrowheads and positions of the longest (1,674 bp) and the shortest (558 bp) OC markers are shown as blue spots. The extra arc of unknown origin is indicated with black arrow on panels A, B, D and E. (A-B) Detection of Sc119 repeats in *Secale cereale*, demonstrating the effect of single-stranded DNA degradation by mung bean nuclease (B) compared to untreated control (A). Figure insets contain magnified regions marked by the rectangles showing the effect of the nuclease treatment on shape of the spots. All other samples shown on panels C-L were also treated with mung bean nuclease. (C) Afa repeats in *Triticum aestivum*, (D) IGS-like in *Vicia sativa*, (E) CentO in *Oryza sativa*, (F) FokI in *V. faba*, (G) PisTR-B in *Pisum sativum *(this sample was resolved on 1.5% and 2% 2-D gels), (H) VicTR-B in *V. grandiflora*, (I) VG-V subfamily of VicTR-B in *V. grandiflora*, (J) VicTR-A in *V. pannonica *detected using PCR probe including a mixture of genomic VicTR-A sequences, (K-L) VicTR-A in *V. pannonica *detected using specific clones c653 (K) and c666 (L).

Extrachromosomal circular molecules were detected for all nine satellite families and three subfamilies tested, and in all cases they occurred as open circles (Fig. 3). The size of the eccDNA molecules ranged from over 8 kb down to 500 bp and in most cases their hybridization signals formed discrete spots corresponding to multiples of monomer or higher-order unit lengths (Table 1). However, there was often a continuous smear underlying these spots, as shown on Fig. 3A for Sc119 satellite of *S. cereale*. Treating the samples with mung bean nuclease, which specifically degrades single-stranded but not double-stranded or nicked DNA almost completely removed the smear, resulting in more round and discrete spots (Fig. 3B), thus suggesting that the smear was composed of partially single-stranded circular molecules. In addition to the arcs representing circular and linear DNA molecules, in some cases we encountered hybridization signals forming a relatively fuzzy arc that could not be assigned to linear, covalently closed supercoiled or open circular molecules by co-migration with the respective markers. The signal was reproducibly detected for satellites from *Secale cereale, Vicia sativa *and *Oryza sativa *(Fig. 3B, D, E). As it was present even in mung bean nuclease-treated samples, its composition from single-stranded DNA or RNA could be ruled out. Thus, we tested whether it could be affected by treatment with RNaseH or RNaseH followed by mung bean nuclease. However, these treatments had no effect, implying that the extra arc did not include hybrid DNA:RNA molecules (data not shown).

The series of discrete spots representing open circles differing in size by the monomer length of the respective satellites were detected for all repeats with monomers ranging from 340 bp (Afa of *Triticum aestivum*, Fig. 3C) down to 118 bp (Sc119 of *S. cereale*, Fig. 3A, B). *V. faba *FokI repeats (monomer size of 59 bp) and all other satellites with shorter monomers produced continuous arcs of hybridization signal (Fig. 3F, G, H), which could also be made up of the monomer-spaced spots that, due to the limited resolution of agarose gel electrophoresis, were fused into a continuous smear, even when 1.5% agarose was used in the first dimension instead of 0.7% (Fig. 3G). However, there were also several repeats showing more complex patterns of eccDNA signals. In the case of the *O. sativa *CentO repeats, the signals formed short smears instead of focused spots (Fig. 3E), which could be explained by the presence of monomer variants differing in sequence length (145, 165 bp) interspersed within the arrays of predominant 155 bp monomers, as reported by Lee et al. [25]. VicTR-B repeats of *V. grandiflora *produced a continuous smear consistent with their short monomer length (38 bp); however, there were also faint spots detected on the smear differing by 186 bp (not shown). Further investigation using specifically selected probe and stringent hybridization conditions revealed that the discrete spots represented a minor sequence subfamily VG-V reported by Macas et al. [26] which is homogenized as a 186 bp higher-order repeat derived from five monomers (Fig. 3I).

**Figure 4:**
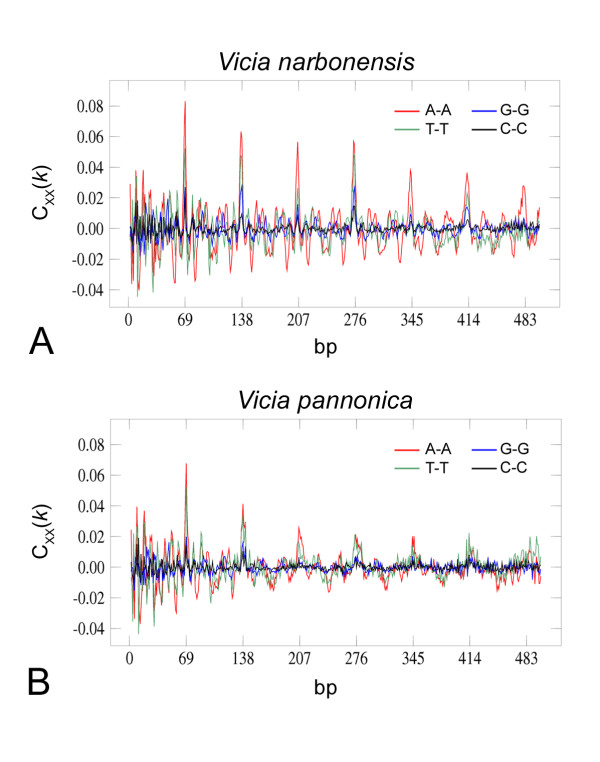
**Periodicity analysis of VicTR-A repeats in *Vicia narbonensis *and *Vicia pannonica***. The nucleotide autocorrelation analysis of VicTR-A sequences from *V. narbonensis *(A) and *V. pannonica *(B), measuring the excess of pairs of identical nucleotides at various distances (2–500 base pairs). Sequence periodicity was calculated for a distance of *k *base pairs and nucleotide X as a difference C_XX_(*k*) = p_XX_(*k*)-p_X_.p_X_, where p_XX _is the observed frequency of identical nucleotides X and p_X _is the proportion of nucleotide X in the sequence [26,27]. The periodicity is revealed as regularly spaced peaks. The results combining periodicities of all four nucleotides are shown.

The VicTR-A satellite of *V. narbonensis *was found to produce eccDNA in size steps corresponding to dimers (138 bp) and more frequently tetramers (276 bp) of the basic repeated unit of 69 bp (Fig. 2). In the related species *V. pannonica*, initial investigation of the VicTR-A repeats, using probe amplified in a PCR reaction with genomic DNA as a template, revealed a different hybridization pattern for their eccDNA, consisting of the smear with spots spaced by about 180 bp which could not be derived from multiples of the monomer length (Fig. 3J). As there was only limited information available about sequence variability of VicTR-A satellites in *V. narbonensis *and *V. pannonica*, we constructed whole genome shotgun libraries from these species and screened them for VicTR-A clones which were subsequently sequenced and analyzed. The analysis of 29 *V. narbonensis *VicTR-A clones (19,006 bp in total) using nucleotide autocorrelation functions [26,27] revealed that their sequences are well-homogenized with a basic periodicity of 69 bp. However, some preference for homogenization of the tetramer-based units was also revealed by the increased height of the 276 bp peak (Fig. 4A). This is consistent with the observed preferential formation of the tetramer-based eccDNA, although it is not clear why similar correlation was not observed for the dimer periodicity, which was detected in eccDNA sizes but not by the sequence analysis.

**Figure 5:**
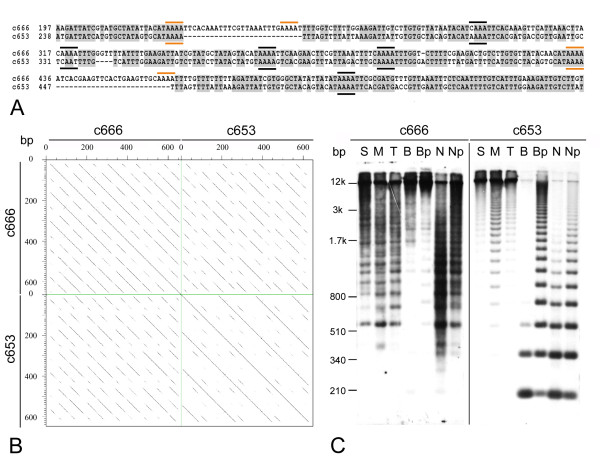
**Sequence similarity and genomic organization of VicTR-A subfamilies in *Vicia pannonica***. (A) Alignment of the clones c666 and c653, representing the subfamilies with 69/138 and 180 bp periodicities. The A-tracts involved in recombination between the 69/138 repeats which presumably gave rise to the 180 bp subfamily are indicated with orange lines, while other A-tracts are marked with black lines. Only a part of the alignment is shown. (B) Dot-plot comparison of the clones c666 and c653, displaying similarities between the sequences as diagonal lines [48]. (C) Southern blot of *V. pannonica *genomic DNA digested with various enzymes and hybridized with probes derived from VicTR-A clones c666 and c653, respectively. Enzymes used for DNA digestion were: S, *Sau*3A; M, *Mbo*I; T, *Taq*I; B, *BsmF*I; N, *Nla*III; Bp, Np, partial restriction digestion of *BsmF*I and *Nla*III, respectively.

In contrast to *V. narbonensis*, analysis of 26 *V. pannonica *clones (16,234 bp in total) showed that they are considerably less homogenized, as revealed by much lower peak heights (Fig. 4B). Their basic periodicity was also 69 bp, however, there was one *V. pannonica *clone (c653) with periodicity of 180 bp and high sequence similarity (> 97%) between the 180 bp repeated units. Comparative analysis with other VicTR-A sequences (Fig. 5A,B) revealed that this repeat most probably originated by recombination between AT-rich regions resembling the CAAAA motif, which is supposed to be involved in breakage-reunion of repeated sequences [28,29]. Using this clone as a probe confirmed that this VicTR-A subfamily gives rise to the 180 bp-spaced signals on the blots of extrachromosomal circular molecules (Fig. 3K), while the other subfamily with 69/138 bp periodicity (clone c666) produces weak signals of continuous smear (Fig. 3L). Southern blots of restriction enzyme-digested genomic DNA hybridized with the probes differentiating the two subfamilies confirmed that the 180 bp subfamily occurs in the *V. pannonica *genome along with the previously reported VicTR-A repeats with 69/138 bp repeated units [3] (Fig. 5C).

## Discussion

Our results show that all the plant satellite repeats that we investigated are prone to the formation of eccDNA. These results complement similar findings described for insects and animals [13-15,30], and significantly broaden our knowledge about plant satellite repeats [16,17] by detection of satellite-derived eccDNA in a total of ten species. Investigated satellites differed in their monomer length, proportion in the genome and chromosomal localization (Table 1). Since eccDNA was detected for all of them, it can be concluded that these features do not have a crucial impact on the formation of circular molecules. A common feature of all investigated satellites was that their eccDNA occurred in the form of open circles, the double-stranded circular molecules relaxed due to the presence of singe strand nicks. The possibility that these findings resulted from DNA damage during sample preparation was excluded by detection of intact supercoiled control plasmid added to the samples at the beginning of the isolation procedure. Similar control was used for eccDNA isolation from *Xenopus *embryos which was also found to occur as open circles [13].

In yeast, eccDNA formation requires chromosomal replication as it originates from stalled replication forks [31]. On the other hand, eccDNA production in *Xenopus *is supposed to be uncoupled from DNA replication, although some synthesis requiring replicative polymerases was detected on the newly formed eccDNA [13,32]. The experiments that focused on FokI repeats in *V. faba *revealed comparable levels of eccDNA in mature leaves and young (growing) leaves or root meristems, thus indicating that FokI eccDNA formation is also not tightly linked to DNA replication. We did not observe a significant increase of eccDNA concentration in mechanically damaged leaves, suggesting that its formation is not induced by this sort of stress or DNA degradation processes. It should be noted, however, that due to generally low levels of eccDNA in the investigated tissues and the only semi-quantitative nature of the assay, we could not detect subtle changes in eccDNA concentration.

Our experimental results, together with previous reports [14,17,32], support the hypothesis that eccDNA is produced by homologous intra-strand recombination between satellite repeat units [11,33]. This process is supposed to result in eccDNA molecule sizes corresponding to the multiples of monomer length, which is consistent with our observations. Moreover, the eccDNA patterns detected for different VicTR-A and VicTR-B subfamilies or higher-order repeats suggest that relatively long regions (tens to hundreds of nucleotides) of high sequence similarity are required for efficient recombination. It has been reported that efficiency of homologous recombination depends on similarity of involved sequences [34-36] and is proportional to the length of the similarity [37]. If the length or degree of sequence similarity is decreased, the rate of recombination is reduced rapidly. In yeast, a divergence of only 1% between 350 bp substrates caused a 5–23-fold reduction of mitotic and meiotic recombination, and divergence of 15% led to a 700-fold reduction [38]. In plants, 1.6% and 1.9% sequence heterogeneity was found to decrease the frequency of intrachromosomal recombination by 3.6 [36] and 9.6-fold [39], respectively, and the recombination between 585 bp inverted repeat substrates was reduced by about 4–20 fold when the level of divergence increased from 0.5% to 9% [40]. A similar adverse effect of decreasing similarity on the efficiency of eccDNA formation was also evident in the case of HOR units of the subfamily VG-V of *V. grandiflora *VicTR-B repeats. The 186 bp HORs have significantly higher average similarity (89%) than the individual monomers from which they are composed (78%) [26]. Consequently, the eccDNA was detected only in sizes corresponding to multiples of the HOR lengths but not in multiples of the 38 bp monomers (Fig. 3I). Other VicTR-B subfamilies from the same species that are homogenized at the level of the monomers produced eccDNA with corresponding size distributions (Fig. 3H). Thus, homologous recombination seems to be the major mechanism of eccDNA origin from plant satellite repeats. Other possible recombination mechanisms such as the nonhomologous end joining (NHEJ) repair pathway probably do not contribute significantly to eccDNA production [16]. This conclusion is also consistent with previous studies on yeast, which demonstrated the requirement for RAD52-dependent homologous recombination in the formation of eccDNA from rDNA repeats [41].

As the eccDNA size distributions of satellites corresponded to multiples of monomer or higher-order repeat lengths we did not find any direct evidence of eccDNA production reflecting the periodicity of nucleosomal structure of chromatin. Nevertheless, it cannot be ruled out that a minor subpopulations of circles arise via this recombination pathway and were not detected under our experimental conditions. Such a mechanism could explain the origin of the VG-V subfamily, which represents a clear example of evolutionary shift from 38 bp monomers to the pentamer-based 186 bp HORs. Moreover, the recombination-based elimination of specific sequence regions was likely involved in the formation of the 180 bp VicTR-A subfamily in *V. pannonica *(Fig. 5A,B) which could represent another case of evolution towards the nucleosome-sized monomers. The constraints imposed on the formation of eccDNA by chromatin could also explain some discrepancies observed when comparing sequence periodicity of *V. narbonensis *VicTR-A satellite (Fig. 4A) to the size distribution of its eccDNA spots detected on 2-D blots (Fig. 2). While the periodicity of the sequenced VicTR-A clones was found to be based on a monomer-sized repeated units, the eccDNA was found to occur in multiples of dimer and preferentially tetramer units. Interestingly, the tetramer peak (276 bp) on the periodicity plot is higher that the one corresponding to the trimer (207 bp), suggesting emergent tetramer-based periodicity. Thus, this satellite might be in a transition stage towards HOR periodicity driven by preferential formation of dimer/tetramer-based eccDNA. On the other hand, we can not exclude that the observed eccDNA pattern could arise from some unknown, less abundant subfamily of VicTR-A repeats with already developed HOR periodicity which we did not detect among the cloned sequences.

The observed common occurrence of eccDNA is important for proving its role in satellite repeat evolution, but it remains to be investigated to what extent it participates in the processes of satellite repeat amplification and sequence homogenization. In the simplest case, recombination-based excision of eccDNA may only represent a deletion mechanism reducing copy numbers of satellite repeats in the genome. Alternatively, open circular molecules can be further utilized as a replication template, leading to production of long linear stretches on newly synthesized DNA fragments composed from multiple copies of the original circular sequence. Thus, this mechanism would provide both amplification as well as sequence homogenization of satellite DNA. In *Drosophila*, specific circle-with-tail structures of tandemly arranged genes corresponding to *Stellate*, *Suppressor *of *Stellate *and histone genes were observed on 2-D gels, suggesting the occurrence of rolling circle replication of these eccDNA. Such rolling circle intermediates (RCIs) of satellite eccDNA molecules were not, however, observed due to the methodological constraints of 2-D electrophoresis [42]. Nor did we find RCIs of satellite eccDNA in our experimental system. This obstacle could be overcome in future experiments by visualizing the content of samples under the electron microscope, as already successfully done for RCIs of mitochondrial plasmid mp1 in *Chenopodium album *[43] and rDNA rolling circles in *Xenopus *[44].

## Conclusion

This work demonstrated the existence of eccDNA molecules derived from various plant satellite repeats, providing strong support for theoretical models predicting eccDNA as an intermediate in satellite DNA evolution. However, it is yet to be seen to what extent and how the eccDNA is utilized in these processes. Future detailed examination of the molecular basis for recombination events and analysis of replication intermediates should provide a better understanding of the biological principles and constraints involved in these processes.

## Methods

### Plant material and genomic DNA isolation

Seeds of plants used in this study were obtained from Osiva Boršov, Czech Republic (*V. faba *cv. Merkur, *V. pannonica *cv. Detenická panonská), IPK Gatersleben, Germany (*V. grandiflora *Scop. var. grandiflora, *V. narbonensis *L.), NASC, Loughborough, UK (*Arabidopsis thaliana *Columbia), the Breeding Station at Slapy u Tábora, Czech Republic (*Pisum sativum *cv. Carrera), the Agriculture Research Institute at Kromeříž, Czech Republic (*Vicia sativa *cv. Ebena), and the Crop Research Institute, Prague, Czech Republic (*Secale cereale *cv. Dankovské, *Triticum aestivum *cv. Saxana). Seeds of rice (*Oryza sativa *ssp. *japonica *var. Nipponbare) were kindly provided by Prof. J. Jiang (University of Wisconsin, Madison, USA). Total genomic DNA was extracted from leaves pooled from several plants as described by Dellaporta et al. [45]. In *V. faba*, DNA was isolated from young (developing) leaves, mature (one-month-old) leaves or 0.5 cm long root tips in order to compare eccDNA levels in various tissues. The DNA concentration measurements were performed using PicoGreen dye (Invitrogen, USA) according to the manufacturer's recommendations.

### Preparation of circular DNA size markers

Plasmid-based open circle markers were prepared by cloning *Pst*I-digested lambda DNA (Fermentas International Inc., Canada) into plasmid vector pBluescript II SK+ (Stratagene, USA). Selected plasmid clones of different sizes were isolated and converted from a supercoiled to a open form by nicking activity of DNaseI (Boehringer Mannheim, Germany). The reaction was performed in a mixture consisting of 25 pg of DNaseI, 50 mM Tris-HCl pH 7.5, and 10 mM MgCl_2 _(total volume of 15 μl), for 15 min at 37°C. Open circle markers of small sizes (558, 930, 1,302, 1,674 bp) were designed using LoxP-directed cloning [46]. Complementary oligonucleotides (5'-GAT CTA TAA CTT CGT ATA ATG TAT GCT ATA CGA AGT TAT G-3', 5'-AAT TCA TAA CTT CGT ATA GCA TAC ATT ATA CGA AGT TAT A-3') were annealed to form a linear double-stranded fragment harboring the LoxP site and single-stranded overhangs compatible with *Bam*HI and *Eco*RI restriction sites. The fragment was cloned into *Bam*HI/*Eco*RI-digested plasmid vector and this construct was further modified by incubation with Cre recombinase (New England BioLabs, USA) and the linear LoxP fragment (in a 20 μl reaction mixture containing 2 μg LoxP vector, 0.5 μM annealed LoxP oligonucleotides, 1 × ligase buffer (Fermentas International Inc.), 10 U Cre recombinase, at 37°C for 30 min and heat-inactivated at 70°C for 10 min), producing linearized plasmid carrying one LoxP sequence terminated with a *Bam*HI and *Eco*RI overhang at each end, respectively. This vector was used for cloning *Bam*HI/*Eco*RI-digested PCR fragments of various lengths amplified from the lambda DNA template using a forward primer (5'-TTG CTG AGG ATC CTG TAC CGG CTG TCT GGT ATG TAT G-3') in combination with one of the following reverse primers (5'-TTG CTG AGA ATT CTC CTC CTG CGA TCC CTT C-3', 5'-TTG CTG AGA ATT CAT CGG CAG GGT GAT CGC-3', 5'-TTG CTG AGA ATT CTG GAA CTG GCG AGC CAT C-3', 5'-TTG CTG AGA ATT CGC GGC TTC AAG CGC AAG-3'). The final constructs thus contained LoxP-PCR fragment-LoxP cassettes which were subsequently released by Cre-mediated recombination in the form of covalently closed circular molecules. The length of these circular DNA markers was 558, 930, 1,302 or 1,674 bp. They were treated with a nicking endonuclease Nt.AlwI (New England BioLabs) to convert them into open circles, and purified by agarose-gel electrophoresis. 50 pg of each of these markers were added into genomic DNA samples prior to 2-D electrophoresis and their positions on the gel were determined using Southern hybridization with the lambda DNA probe.

### EccDNA analysis on two-dimensional agarose gel electrophoresis

Neutral-neutral 2-D agarose gel electrophoresis was performed as described by Cohen and Lavi [22] with the following modifications. Samples of up to 20 μg of genomic DNA were analyzed (in the case of comparative analysis of eccDNA content in different tissues or in stressed plants, the same DNA amouts were always loaded). The DNA was separated on 0.4% agarose in 1 × TBE buffer at 0.7 V/cm for 18 hr and the lanes with samples were excised and stained in 1 × TBE buffer containing 0.3 μg/ml of ethidium bromide for 2 hr. Stained lanes were placed on a gel support at 90° orientation to the direction of electrophoresis and embedded by 1% agarose supplemented with 0.3 μg/ml of ethidium bromide. The second dimension was run in 1 × TBE buffer, 0.3 μg/ml of ethidium bromide at 4 V/cm for 4 hr. Alternatively, the electrophoresis was run on 0.7% and 2% agarose gels (in some cases, 1.5% and 2% was used) for 21 and 8 hours, respectively, in order to improve resolution of small DNA molecules. In addition, the sensitivity of the assay was increased by treating the samples with Plasmid-safe ATP-dependent DNAse (Epicentre Biotechnologies), which selectively degraded linear DNA fragments, thus allowing equivalents of 80 – 240 μg of undigested genomic DNA to be loaded. The Plasmid-safe ATP-dependent DNAse treatment was preceded by passing high molecular weight genomic DNA through a hypodermic needle (Omnican 100, 0.3 mm in diameter, B. Braun Petzold GmbH, Germany), resulting in its slight shearing which promoted linear DNA degradation. The treatment was performed using 160 – 480 U of Plasmid-safe ATP-dependent DNase in 800 – 2400 μl of reaction buffer overnight at 37°C and stopped by incubation at 70°C for 30 min. Short fragments of degraded nucleic acids were removed using molecular weight cut-off columns (Microcon-30 and Microcon-100, Millipore, USA) and the digestion and purification was repeated once more.

The presence of single-stranded DNA was tested by dividing purified samples into halves and incubating them with or without 5 – 50 U of mung bean nuclease (MBN, Takara Bio Inc., Japan) in 1 × MBN buffer for 10 min at 37°C. Alternatively, RNaseH (Ambion, USA) directed degradation or a combination of RNaseH followed by MBN treatment was performed to check for the presence of DNA-RNA hybrid molecules. RNaseH treatment was carried out in 100 μl of 1 × RNaseH reaction mixture and 25 U of RNaseH for 1 hr at 37°C. Finally, to remove any contamination that could affect subsequent electrophoretic separation, the samples were purified using Wizard SV Gel and PCR Clean-Up System columns (Promega, USA).

### Southern hybridization

Following electrophoresis, DNA was transferred onto Hybond-N+ membranes (Amersham Biosciences, USA) by capillary transfer. Hybridization probes for satellite repeats were derived from fragments amplified by PCR using specific primers and genomic DNA as a template (AT_180: 5'-ACC TTC TTC TTG CTT CTC AAA G-3', 5'-GTT GGT TAG TGT TTT GGA GTC G-3'; CentO: 5'-AAA ACA TGA TTT TTG GAC ATA TTG G-3', 5'-TGA CAA AAG TTC GCC GCC-3'; PisTR-B: 5'-ACC CAT GAA ATT TGA TTG-3', 5'-CAA CAT TTT CAT CAT TCA CAC-3'; Afa: 5'-GCA TTT CAA ATG AAC TCT GA-3', 5'-GAT GAT GTG GCT TTG AAT GG-3', Sc119: 5'-CCA GAA TCG GCC AAA AC-3', 5'-CCC GTT TCG TGG ACT ATT AC-3'; FokI: 5'-CAT TAT GGA AGG TAG TCT GTT GTC GAG-3', 5'-CAA GGC TAC CAT CCA TTG GAG-3'; VicTR-A: 5'-TAC ATA AAA GTC AYG AAG TT-3', 5'-TAS TAT AAC AYA AGA YA ATC-3'; VicTR-B: 5'-ATA TAA GTC TTC ARA AAA T-3', 5'-GAA GAC TTA TAT TCA CTT-3'). The probe for IGS-like satellite was prepared by insert amplification from clone S12 [47] using T3/T7 primers and removing surrounding polylinker sequences by restriction digestion and gel purification. The same procedure was used for probe preparation for the VG-V subfamily of VicTR-B repeats (using clone c609 [GenBank:DQ139394]) and for VicTR-A clones c653 [GenBank:EU568805] and c666 [GenBank:EU568818]. Fragment labeling and hybridization were done using the AlkPhos Direct Kit (Amersham Biosciences), according to the manufacturer's recommendations (hybridization and washing temperatures varied between 50°C and 61°C according to probe AT/GC content). Hybridization specificity for the VG-V subfamily was verified in hybridization using clone c609 as a probe and membranes with clones c605 [GenBank:DQ139381], c788 [GenBank:DQ139383], c606 [GenBank:DQ139368] and c610 [GenBank:DQ139388] [26] representing other VicTR-B subfamilies. The specificity of VicTR-A clones c653 and c666 was confirmed in hybridization using sequences of clones c651 [GenBank:EU568803], c652 [GenBank:EU568804], c654 [GenBank:EU568806], c763 [GenBank:EU568830] and c768 [GenBank:EU568835] as negative controls. To detect signals, blots were incubated with chemiluminescent substrate (CDP-Star, Amersham Biosciences) and exposed to X-ray film for up to 60 hr.

### Cloning and sequence analysis of VicTR-A repeats

The preparation of *V. pannonica *and *V. narbonensis *shotgun genomic libraries and screening and sequencing of their clones was performed as described by [26] except that VicTR-A clone P5 [3] was used as a probe to screen the libraries by colony hybridization. The sequences were deposited in GenBank under the accession nos. EU568802 – EU568868. Sequence periodicity analysis based on the concept of nucleotide autocorrelation functions [27] was performed as described previously [26]. Dot-plot sequence comparisons were done using a dotter program [48].

## Authors' contributions

AN and JM designed the study, and AN carried out most of the experimental work. AK constructed and screened genomic DNA libraries and participated in eccDNA detection experiments. JM performed bioinformatic analysis of the newly sequenced satellite repeats. All authors contributed to the manuscript preparation and approved its final version.
